# Vaccines for *Streptococcus agalactiae*: current status and future perspectives

**DOI:** 10.3389/fimmu.2024.1430901

**Published:** 2024-06-14

**Authors:** João Matheus Sobral Pena, Pamella Silva Lannes-Costa, Prescilla Emy Nagao

**Affiliations:** Laboratory of Molecular Biology and Physiology of Streptococci, Institute of Biology Roberto Alcantara Gomes, Rio de Janeiro State University - UERJ, Rio de Janeiro, Brazil

**Keywords:** Streptococcus agalactiae, vaccines, maternal immunization, immunogenicity, vaccine acceptance, cost-effectiveness

## Abstract

A maternal vaccine to protect newborns against invasive *Streptococcus agalactiae* infection is a developing medical need. The vaccine should be offered during the third trimester of pregnancy and induce strong immune responses and placental transfer of protective antibodies. Polysaccharide vaccines against *S. agalactiae* conjugated to protein carriers are in advanced stages of development. Additionally, protein-based vaccines are also in development, showing great promise as they can provide protection regardless of serotype. Furthermore, safety concerns regarding a new vaccine are the main barriers identified. Here, we present vaccines in development and identified safety, cost, and efficacy concerns, especially in high-need, low-income countries.

## 1 Introduction

*Streptococcus agalactiae* is one of the main causes of invasive infections in neonates, elderly, and adults with comorbidities. The main risk factor for *S. agalactiae* infection in newborns is maternal rectovaginal colonization during pregnancy, which *S. agalactiae* can cause intrauterine infection, premature labor and/or stillbirth (1). Approximately 20 million pregnant women worldwide were colonized by the microorganism in 2020 and nearly 400,000 children suffered from early-onset *S. agalactiae* disease (EOD, 0 to 6 days after birth) or late-onset disease (LOD, 7 to 89 days after birth). In addition, there were 90,000 child deaths, almost half of which were in Sub-Saharan Africa. Approximately 46,000 stillbirths resulting from *in utero S. agalactiae* infection and more than 500,000 preterm births may have been associated with *S. agalactiae* colonization in 2020 (2). The numerous negative effects resulting from maternal *S. agalactiae* colonization imply the need for an effective prevention approach that can reduce the risk of multiple outcomes.

High-income countries screen pregnant women colonized by *S. agalactiae* at the end of the third trimester, and intrapartum antibiotic prophylaxis (IAP) is administered to those who test positive. However, this conduct is not practiced in middle- and low-income countries. IAP is effective in preventing EOD (~ 80%) but is not effective against LOD or prenatal sequelae associated with *S. agalactiae* infection. Neonates with EOD have greater susceptibility to respiratory diseases, seizures and long periods of hospital stay (3). Additionally, individuals who survive may suffer uncontrolled seizures, intellectual disability, deafness, blindness, and impaired psychomotor development (4).

Maternal vaccination against *S. agalactiae* has been proposed by the World Health Organization (WHO) as an alternative preventive strategy based on the immunological protection provided by placental passage of maternal immunoglobulin G (IgG). In this way, there would be protection both against EOD and LOD, also against complications associated with puerperal disease. Over the past two decades, *S. agalactiae* simple capsular polysaccharide (CPS) has been studied as vaccines in preclinical and clinical studies, including the six most prevalent capsular types (Ia, Ib, II, III, IV and V), but have shown that the purified native polysaccharide was not sufficient to induce a robust IgG response in adults. In this way, the conjugation of poorly immunogenic microbial CPS to protein transporters, such as chemically detoxified diphtheria and tetanus toxin (TT) or genetically detoxified diphtheria toxin (CRM197), triggered an anti-carbohydrate T cell memory response (5). Currently, efforts have also focused on identifying protective protein antigens present in most strains of *S. agalactiae*, including non-encapsulated ones.

## 2 Vaccine candidates

**Figure 1:**
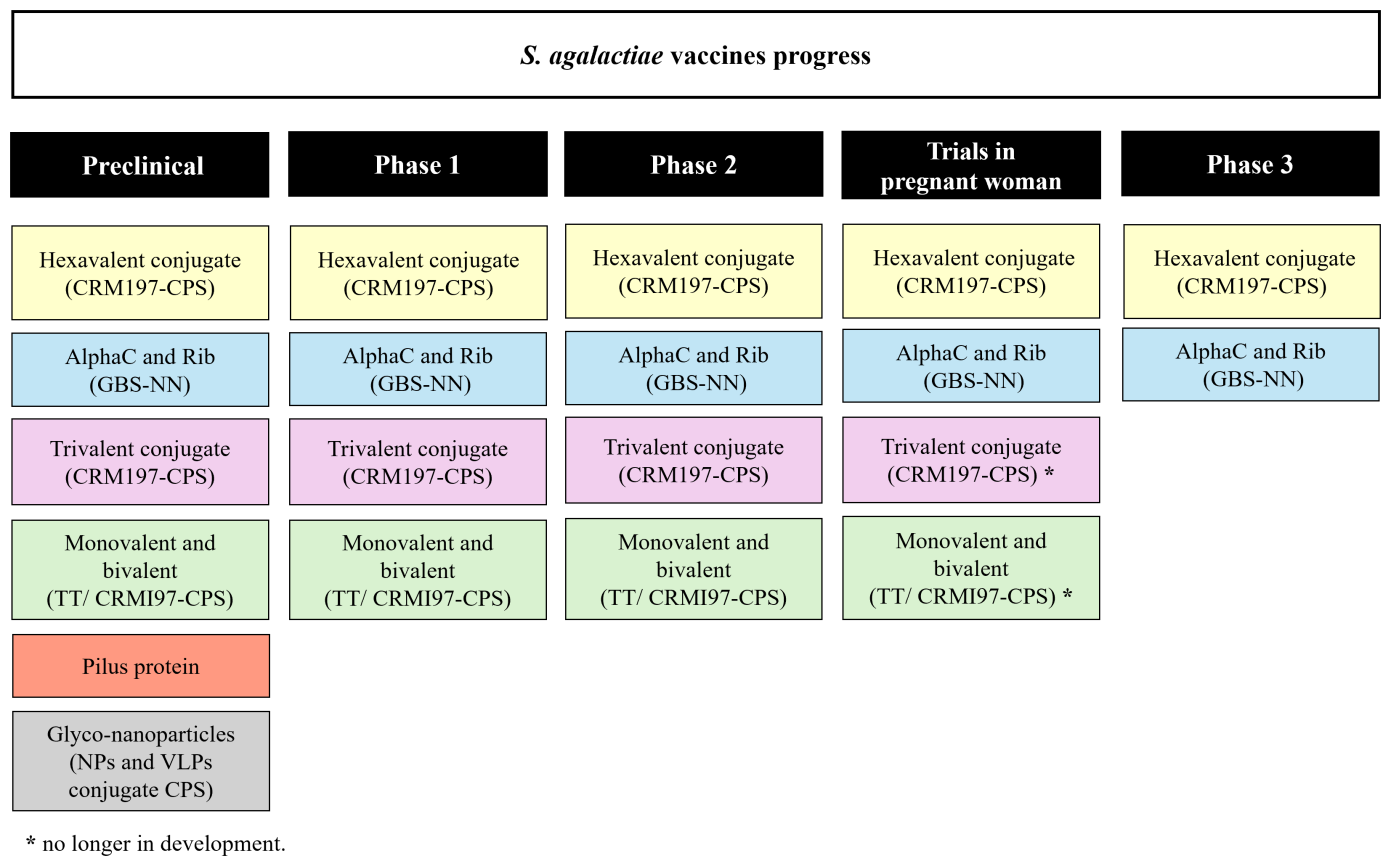
*S. agalactiae* vaccines. Schematic representation of *S. agalactiae* candidate vaccines in preclinical and clinical trials.

The target population of a *S. agalactiae* vaccine in pregnant women is universal with no differentiation between high- and low-income settings. Two vaccine development approaches have been prioritized: vaccines based on capsular polysaccharides and protein-based vaccines (**Figure 1**). The first clinical trials carried out with monovalent and bivalent capsular polysaccharide vaccines conjugated to tetanus toxoid that proved to be safe and well tolerated (2). A trivalent vaccine (Ia, Ib and III) conjugated to CRM197 has also been evaluated in clinical studies with pregnant and non-pregnant women in Belgium, Canada, South Africa and Malawi, showing an acceptable safety profile and induction of immune responses to vaccine serotypes, as well as transplacental transfer of antibodies to their babies (6). Phases 1/2 clinical trial of trivalent vaccine conjugated to CRM197 in pregnant women reported higher levels of specific antibodies against the serotypes tested in newborns and no security concerns (7). However, the monovalent, bivalent conjugates (TT and CRM197-CPS) and trivalent CRM197-CPS vaccines are no longer in development (2, 8).

Subsequently, in 2017, a clinical trial with a pentavalent (Ia, Ib, II, III and V) CRM197 conjugate vaccine was initiated. Although, with the emerging invasive *S. agalactiae* disease by serotype IV, this serotype was included to create a hexavalent vaccine (Ia, Ib, II, III, IV and V) with the aim of covering approximately 98% of *S. agalactiae* invasive disease (9). Multivalent capsular polysaccharide vaccines include candidates in various stages of development. Pfizer designed a hexavalent vaccine (GBS6) to target these serotypes; phase 2 evaluation to assess the safety and immunogenicity in pregnant women is currently underway and phase 3 trials started in 2023 (2).

Potential vaccines containing protein subunits have also been analyzed, they may cover different serotypes of *S. agalactiae* and address concerns about serotype replacement or capsular switching (10). The vaccine developed by Minervax, utilizing the N-terminal domain of the alpha-like surface protein family (GBS-NN), has been found to be safe and immunogenic in non-pregnant women and pregnant women (2). Members of the Alp family (AlphaC, Alp1–4 and Rib) are the most well-known and abundant surface proteins. These proteins are expressed by different serotypes of *S. agalactiae* (11). The vaccine GBS-NN displays good safety and immunogenicity profiles in a randomized, placebo-controlled, double-blind phase 1 study involving 240 vaccinated adult healthy women (12).

*S. agalactiae* also expresses either of the two-allelic serine-rich repeat 1 (Srr1) and serine-rich repeat 2 (Srr2) proteins, both of which can bind fibrinogen, contributing to the pathogenesis of meningitis and colonization on the vaginal surface by *S. agalactiae* (13). Results in murine models demonstrated serotype-independent protection against *S. agalactiae* infection after being vaccinated with the latch peptide vaccine. In the case of *S. agalactiae*, Srr1 and Srr2 can bind to the fibrinogen through the dock, lock, and latch mechanisms. After bound, the C-terminal ends of the glycoproteins, which contain a domain known as the latch, undergo a conformational change to obstruct the ligand. These findings indicated that the latch domain of Srr might constitute an effective peptide vaccine candidate for *S. agalactiae* (14). Another potential vaccine candidate or transporter for *S. agalactiae*-CPS is the peptidase C5a, an important virulence factor of *S. agalactiae* (15). C5a peptidase encapsulated within microspheres composed of lactic acid and glycolic acid copolymer triggered systemic and mucosal immune responses in murine models, thereby protecting them against multiple serotypes (16). Additionally, as the role of pili is to promote bacterial adhesion to host tissue, it has become tempting to speculate that pili-based vaccines may also produce antibodies capable of preventing colonization by *S. agalactiae*. The pilus-based vaccine may be effective in preventing infections and capable of providing broad protection by inducing immune responses against different *S. agalactiae* serotypes. However, due to their antigenic variation not all protective pilin subunits can be included in the vaccine (17).

Another vaccine alternative is the conjugation of CPS with protein nanoparticles (NPs) or virus-like particles (VLPs). NPs and VLPs are systems that improve the uptake and activation of immune cells due to their size and dense antigen display (18). In 2023, Carboni and colleagues obtained and optimized self-assembling virus-like particles conjugated to *S. agalactiae* CPS-II, resulting in a glyco-nanoparticles elicited strong immune responses in mice already after one immunization, providing pre-clinical proof of concept for a single-dose vaccine (19).

## 3 Licensure for *S. agalactiae* vaccines

To date, no paths to licensing vaccines against *S. agalactiae* have been agreed by regulators. Programs to conduct trials of a vaccine against *S. agalactiae* are underway to provide the evidence necessary to establish immunological correlates of protection that are robust enough to support licensure of the vaccine, including in low- and middle-income countries. Characterization of the safety and immunogenicity profile of a vaccine against *S. agalactiae* (phases 1 and 2) should initially recruit non-pregnant women of childbearing age to determine the ideal dose of the vaccine, need for adjuvant and the vaccination schedule; subsequently, trials should continue in pregnant women (20). Randomized, double-blind, placebo-controlled trials with a specific and well-defined primary endpoint should provide the strongest evidence for vaccine efficacy to support licensure. The issues of a randomized clinical trial are fully discussed by the WHO (21). However, a phase 3 trial for a vaccine candidate against *S. agalactiae* will require 40,000–180,000 woman-infant dyads (22). Therefore, alternative licensing pathways may be considered, with a potentially crucial role for studies that can identify robust correlates of protection/biomarkers associated with risk reduction.

The WHO highlighted 15 steps to facilitate and speed up the vaccine licensing process. In 2021, WHO and the Bill & Melinda Gates Foundation planned a series of “Full Value of Vaccine Assessments” summarizing the key epidemiological features of *S. agalactiae* disease (23). Pre-licensure clinical studies should also evaluate potential interference with other vaccines administered during pregnancy and with the routine schedule of the immunization program, especially for polysaccharide-protein conjugate vaccines administered on the routine schedule such as *Haemophilus influenzae* type b, meningococcal and pneumococcal (24).

Immunogenicity studies indicate that vaccine efficacy may be influenced by co-infection, for example, HIV and syphilis (25) and the prevalence of *S. agalactiae* colonization. However, vaccination only against specific types increases selective pressure, leading to capsular exchange post-vaccination or capsular replacement, and increase in non-encapsulated *S. agalactiae* strains (26, 27). Substantial replacement has been observed after the introduction of pneumococcal conjugate vaccines (28), but not after vaccines meningococcal or *Haemophilus influenzae* type b (29). Thus, strong post-licensure surveillance in several different settings will need to be carried out to address these potential problems.

Licensure of hexavalent vaccine by a pathway requiring conduct of an efficacy study would be complex and likely require many years to complete. In this way, approval based on the protective immunological response may be viable and offer advantages for a faster path to obtaining licensing.

## 4 Immunogenicity

Vaccines under development are designed to provide broad coverage against the main disease-causing serotypes of *S. agalactiae*. Effects on immunogenicity may depend on carrier proteins but are unpredictable and must be studied before widespread use. Conjugated vaccines against *S. agalactiae* appear to induce antibodies that facilitate killing of the bacteria by opsonophagocytosis, but a protective titer of opsonophagocytosis has not yet been defined. However, extrapolation of protective antibody thresholds must be cautious because co-infection with other microorganisms may impair the transfer of placental antibodies (30).

**Figure 2:**
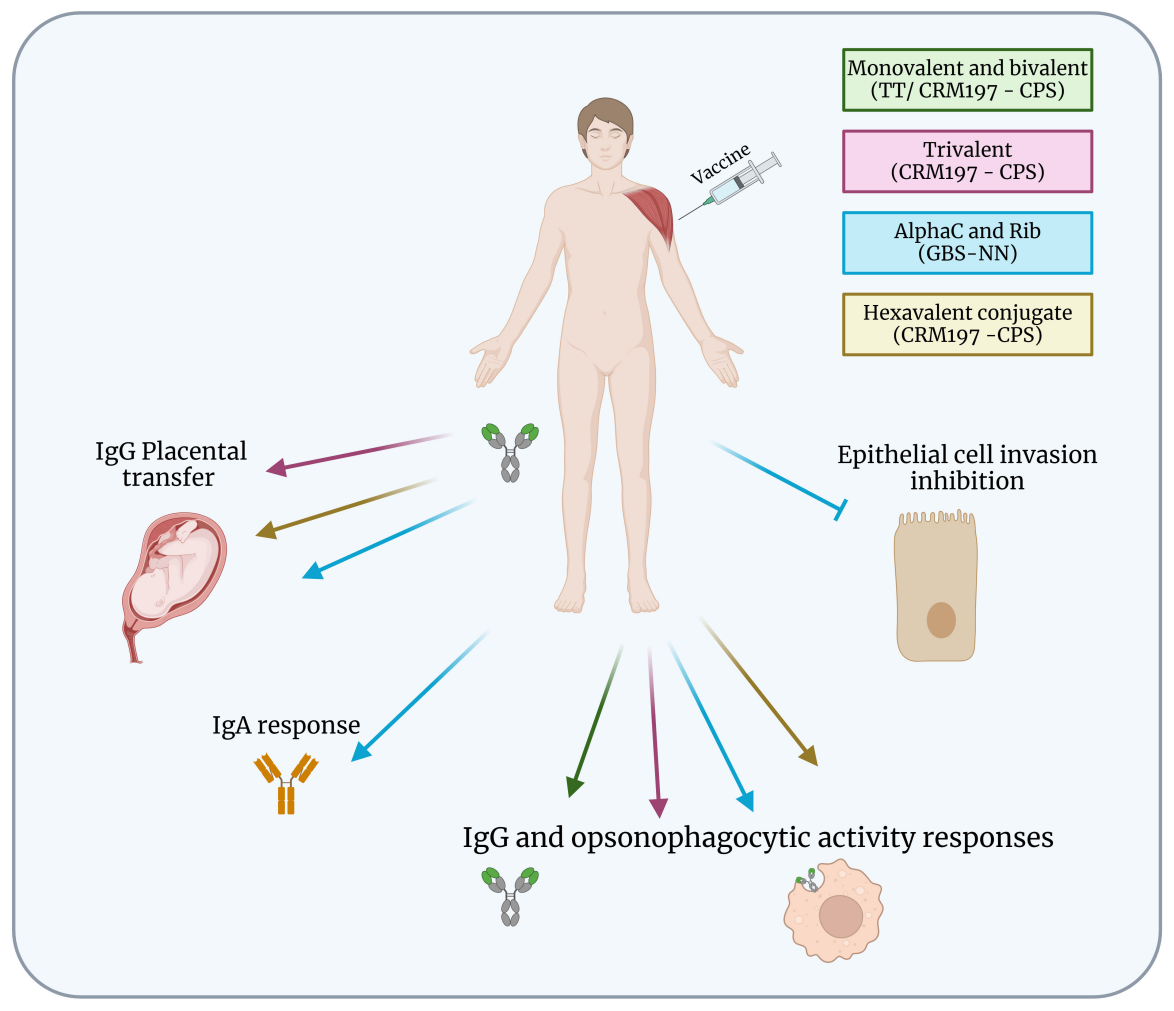
Protection of *S. agalactiae* vaccines in clinical development. Summary of immunogenicity of *S. agalactiae* vaccines in human clinical development. Monovalent and bivalent vaccines induced IgG and opsonophagocytic activity responses but are no longer in development. Trivalent, hexavalent and GBS-NN vaccines induced IgG placental transfer, and IgG and opsonophagocytic activity responses. GBS-NN also triggered IgA response and inhibition of epithelial cell invasion. Figure created with BioRender.com.

Monovalent and bivalent capsular polysaccharide vaccines were able to induce IgG production and opsonophagocytic activity responses that remained above baseline 2 years after immunization (8, 31). The phase 2 of trivalent vaccine demonstrated maternal antibodies production and bacterial killing by opsonophagocytosis. Analysis of umbilical cord sera also revealed a strong correlation between IgG concentrations and opsonophagocytic killing, which was predictive of functional activity against *S. agalactiae* infection (7, 32) (**Figure 2**). However, few studies have evaluated the number of doses that will be required per pregnancy for full immunity. Another study in healthy nonpregnant women demonstrated the safety and immunogenicity of a second dose of the trivalent vaccine in non-pregnant women over a period of 4 to 6 years after administration of the first dose with ≥ 200-fold increased levels of antibodies against *S. agalactiae* after a second dose. Women who had undetectable antibody levels after the first dose also had an increase in anti-*S. agalactiae* concentrations after a second dose (6). These results suggest that additional doses may be needed in subsequent pregnancies. However, after the hexavalent vaccine, monovalent, bivalent, and trivalent vaccines were no longer tested.

Currently, a phase 1/2 clinical trial is being conducted in South Africa to evaluate the safety, tolerability, and immunogenicity of the hexavalent vaccine (GBS6) in healthy pregnant and non-pregnant women (33) (**Figure 2**). Antibody concentrations varied between serotypes, with the highest concentration for serotype Ia and the lowest for serotypes Ib and V. The GBS6 vaccine demonstrated safety and IgG responses were sustained through delivery during birth and transferred to babies and may provide additional benefits for prevention of invasive infections, including sepsis that can occur after birth. The optimized conjugates were immunogenic, alone and in combination, in mice and rhesus macaques, inducing IgG antibodies that mediated opsonophagocytic killing. Active immunization of murine dams with GBS6 prior to mating resulted in serotype-specific protection of pups from a lethal challenge with *S. agalactiae* (9).

Although the GBS-NN vaccine elicits strong and opsonophagocytic antibody responses in healthy adult non-pregnant women, protective thresholds in relation to neonatal disease remain to be established for blood and umbilical cord blood to verify the levels and specificity of antibodies transferred to newborns (**Figure 2**). Despite the results demonstrating that GBS-NN also elicits functional antibodies against these domains in subjects with pre-existing immunity, inclusion of Alp1-N and Alp2/3-N in the vaccine formulation will be required to support robust responses in all serotypes (34). The GBS-NN elicited placentally transferable IgG, and the antibodies mediated opsonophagocytic killing of homotypic and heterotypic strains and prevented *S. agalactiae* invasion of epithelial cells (34, 35). GBS-NN also increased IgA response developed mostly in mucosa-associated lymphoid tissues, and it seems possible that the strong association between vaccine-induced and pre-existing IgA was due the reactivation of IgA+ memory B cells originating from mucosal tissues (36). However, the importance of the IgA response elicited by parenteral immunization with GBS-NN requires future studies.

Previous study with GBS80 pilus protein conjugated with CPS type II, obtained by tyrosine directed ligation, demonstrated good ability to provoke murine antibodies that mediate opsonophagocytic death. Furthermore, it provided protection in newborn mice against *S. agalactiae* infection after vaccination of their mothers (37). Interestingly, another study using a single dose of the vaccine conjugating CPSII with the bacteriophage Qβ viral like coat protein (Qβ VLP) induced IgG and showed opsonophagocytic activity in mice. The CPSII-Qβ induced responses post-1 responses that were superior to those obtained after two doses of CPSII-CRM197, confirming the Qβ VLP as a system that improves responses against immunogenic saccharide targets. Furthermore, IgG and opsonophagocytic activity induced by the CPSII-Qβ conjugate reached a maximum at day 42 and persisted until day 134. Similar data were observed using Qβ carriers with capsular polysaccharide Ia, suggesting that the same approach could be applicable and tested for the other capsule types (19).

Therefore, studies of conjugate vaccines for *S. agalactiae* have shown good correlation between the ability of the vaccine to induce opsonophagocytic IgG antibodies *in vitro* and vaccine protective efficacy against the pathogen in animal challenge experiments (38).

## 5 Cost-effectiveness of *S. agalactiae* maternal vaccination

Previous studies with *S. agalactiae* have shown that immunoglobulin G (IgG) antibodies could be transferred across the placenta, making vaccines a promising method for protecting pregnant mothers, fetuses, and newborns. Although conjugate vaccines have been instrumental in reducing the burden of disease, they are among the most technically challenging and expensive vaccines to manufacture (35). Furthermore, the chemical approaches used to conjugate capsular polysaccharides to carrier proteins result in significant heterogeneity with potential destruction of polysaccharide epitopes and/or transport protein (35). This increases batch-to-batch variability, complicating chemistry, manufacturing, control activities and requiring intense quality control and regulatory scrutiny, resulting in high slow development costs and timelines for conjugate vaccines.

Data from South Africa concluded that vaccination against *S. agalactiae* would prevent 30 - 54% of cases of infections in babies. Vaccines priced at $ 10 to $ 30 with average efficacy could avoid spending $ 676 to $ 2,390/disability-adjusted life year. Furthermore, vaccination associated with intrapartum prophylaxis could prevent 48% of cases at a cost of $ 664 to $ 2,128/per disability-adjusted life-year (33). Study carried out in West Africa showed that the hexavalent vaccine could prevent 55% cases of *S. agalactiae* disease and more than 700 years of life associated with disability, compared to the standard without interventions to prevent disease from the bacteria. A vaccine effectiveness of 70% would cost a total of $ 12. The incidence of *S. agalactiae* proved to be the most influential parameter in the cost-effectiveness relationship, demonstrating once again the importance of the vaccine (39).

Trotter and colleagues estimated that vaccinating approximately 99 million pregnant women in several countries could cost $ 1.7 billion. However, there would be savings of approximately $ 300 million in costs for treating acute infections and $ 85 million in costs directed toward long-term health care for pregnant women and newborns. Overall, vaccination could prevent about 127,000 cases of EOD and 87,300 cases of LOD, thereby preventing 31,000 childhood deaths and 17,900 cases of moderate and severe neurological disease. Furthermore, 23,000 stillbirths due to GBS could be avoided and 185,000 premature births could be avoided. In this way, recent progress in implementing the vaccine against *S. agalactiae* promises to reduce the high rate of mortality and morbidity, especially in places of higher incidence and poverty, where clinical and laboratory services can be precarious (40).

## 6 Discussion and future directions

Invasive *S. agalactiae* disease remains an important cause of infant morbidity and mortality, for which the development of an efficacious vaccine remains a global health imperative. After several decades of research into the protective correlates and immunobiology of *S. agalactiae* CPS, the prospect of the first conjugate vaccine becoming available is now on the horizon with the development of hexavalent vaccine.

In high income countries where IAP is the established standard of care, a *S. agalactiae* vaccine may be used as an adjunct public health tool to reduce residual cases of EOD not addressed by IAP, and to prevent LOD where IAP has not had an impact. Easy deployment of a maternal *S. agalactiae v*accine in low- and middle-income countries, integrated as part of existing private market and national immunization programs, could provide substantial benefits for reducing invasive *S. agalactiae* disease in the beginning of life. Moreover, due to the increase in antimicrobial resistance, a vaccine against *S. agalactiae* could, for the most part, replace IAP. However, a global discussion is needed to evaluate additional evidence and information that is being generated, translating into actions and policies that will allow the development of a vaccine against *S. agalactiae* optimized for use in low-resource settings, as well as managers to prepare their countries for the introduction of the vaccine against *S. agalactiae* in pregnant women. Evidence to support licensure based on studies of immunogenicity and correlates of protection, as well as financial sustainability analyzes could increase the attractiveness of developing vaccines against *S. agalactiae* by manufacturers.

The ability to specifically glycosylated carrier proteins in sequences significantly reduces product heterogeneity, allowing known T cell epitopes to be preserved are strategies that are underway as a future perspective using bioconjugates. Bioconjugates also avoid the need for separate culturing and purification of CPS and carrier proteins, thus reducing the number of release controls, which ultimately reduces production costs (41). In fact, the reduced cost of producing bioconjugates could open the door to new vaccines, especially in high-need, low-income countries that have been overlooked due to the high cost of chemical conjugates. Nanoparticles covered with protein antigens also showed promise for use in bacterial vaccines. NPs have provided new pathways for vaccine delivery as large oligomeric structures, which can display multiple antigens in a single particle, facilitating their uptake by antigen-presenting cells and improving immune responses.

Furthermore, there are several unknowns that must be in the future prospects for the implementation of a safe and effective vaccine, such as: (i) determining the number and timing of doses for optimal coverage during pregnancy, (ii) the number of doses necessary for full protection, (iii) special attention to the regional distribution of serotypes in low- and middle-income countries, (iv) analysis of placental transfer of vaccine-induced immune responses in populations infected with HIV, malaria, syphilis that are prevalent in countries with low- and middle-income families and, (v) alteration of the immunological response and transfer of antibodies across the placenta in mothers co-infected with microorganisms that promote reduced immunity.

## Data availability statement

The original contributions presented in the study are included in the article/supplementary material, further inquiries can be directed to the corresponding author/s.

## Author contributions

JP: Conceptualization, Writing – original draft, Writing – review & editing, Data curation, Investigation. PL-C: Investigation, Writing – original draft, Writing – review & editing, Formal analysis, Funding acquisition, Visualization. PN: Conceptualization, Formal analysis, Funding acquisition, Resources, Supervision, Visualization, Writing – original draft, Writing – review & editing.
